# The role of intelligent technology in the development of urban air mobility systems: A technical perspective

**DOI:** 10.1016/j.fmre.2023.08.006

**Published:** 2023-09-25

**Authors:** Yang Liu, Cheng Lyu, Fan Bai, Omkar Parishwad, Ying Li

**Affiliations:** a Department of Architecture and Civil Engineering, Chalmers University of Technology, Gothenburg SE-41296, Sweden; b Chair of Transportation Systems Engineering, Technical University of Munich, Munich 80333, Germany; c School of Information Engineering, Chang'an University, Xi'an 710010, China

**Keywords:** Urban air mobility, Intelligent technology, Artificial intelligence, Air traffic development, Disruptive transportation mode

## Abstract

Urban Air Mobility (UAM) is an emerging transportation system that aims at revolutionizing urban mobility through the deployment of small electric vertical takeoff and landing (eVTOL) aircraft. The development of UAM is largely driven by advances in Intelligent Technology (IT). This review article provides an overview of the UAM system and discusses the application of IT in UAM. Major challenges facing UAM are also identified, and an outlook on the future of this promising transportation system is presented. Our main conclusions suggest that IT is a fundamental driver of UAM, enabling a range of applications such as air traffic management and autonomous drone control. However, the UAM system is facing a number of challenges, including eVTOL technology, system integration issues, and noise pollution. Despite these challenges, the future of UAM appears promising; as a disruptive transportation mode, UAM is expected to play an important role in addressing the growing demand of urban transportation in the coming decades.

## Graphical abstract

**Figure:**
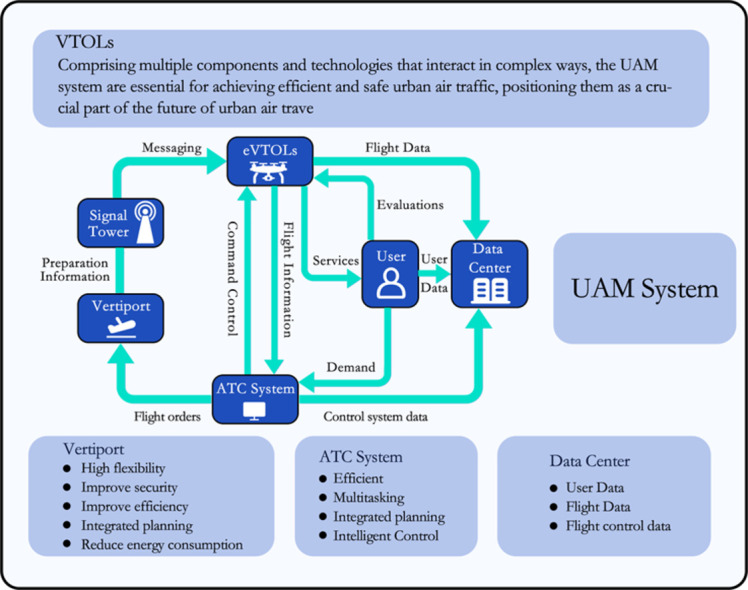

## 1 Introduction

With population growth, accelerated urbanization and economic development, severe urban traffic congestion and environmental deterioration have become significant challenges for major cities worldwide [1,2]. Traditional automobile-oriented urban traffic management and ground transportation modes are no longer adequate to satisfy people's escalating travel demands [3]. Against this backdrop, we are impelled to rethink and adapt our current strategies for traffic management and ground transportation. A new urban mobility solution is required, and the advancement of autonomous driving technologies and the exploration of innovative alternatives are facilitating this transformation [4], [5], [6], [7]. Urban Air Mobility (UAM) emerges as a promising response. The UAM evolution trajectory begins with drones for urban logistics, evolves to electric vertical takeoff and landing (eVTOL) aircraft for passenger commuting, and ultimately anticipates fully autonomous urban flying vehicles [2]. While drones have mitigated certain urban logistics challenges, they do not fit in passenger transport. Notwithstanding the minimal infrastructure reliance of flying vehicle, their technical requirements are still in an early stage of development. Thus, eVTOLs stand out as the immediate and most feasible solution, which will be the primary UAM focus in this paper.

**Fig. 1:**
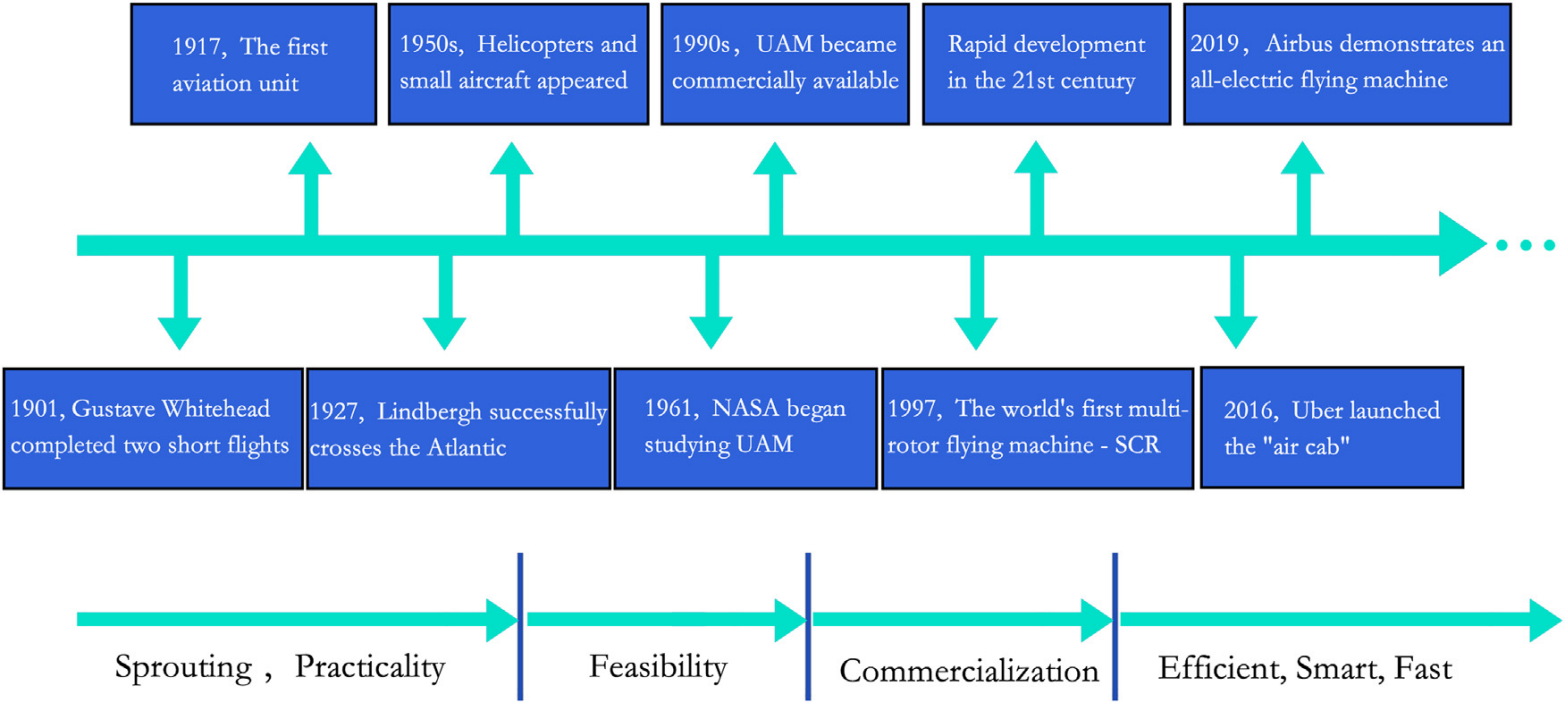
**The evolution of the UAM system**.

In addition to addressing ground traffic issues, UAM also holds the potential to redefine the urban transportation ecosystem, offering safe, efficient, and environmentally-friendly travel alternatives. The historical development of UAM dates back to the early 20th century when flying vehicles were first considered for urban transportation (see Fig. 1). During the 1940s and 1950s, helicopters emerged as a promising means of inter-city travel. However, their widespread adoption was hindered by high costs, excessive noise, and safety concerns [8,9]. Nonetheless, with continuous advancements in technology, particularly in Intelligent Technology (IT), modern UAMs have evolved to offer comfort, affordability, safety, and environmental sustainability. Numerous cities and companies have already initiated research on UAM's practical applications, aiming to explore its potential and development as a complementary public transportation option in the future [10], [11], [12], [13].

IT encompasses a diverse array of emerging technologies. Key developments, such as artificial intelligence (AI) and machine learning, edge cloud systems, and 5G mobile communications, play crucial roles in advancing technologies related to the UAM system and exploring new business models for UAM [14], [15], [16], [17]. The application of IT in UAM can help achieve more efficient, safer and sustainable transportation modes, providing the UAM system with unique advantages in terms of technical characteristics. Specifically, Yang and Wei [18] proposed a computational guidance algorithm with collision avoidance capability to ensure safe and efficient autonomous on-demand free flight operations in UAM. The problem is formulated as a Markov Decision Process (MDP) and solved using an online algorithm Monte Carlo Tree Search. The proposed algorithm was evaluated using a high-density free-flight airspace simulator and revealed fewer conflicts and near-air collisions compared with state-of-the-art collision avoidance strategy, i.e., Optimal Reciprocal Collision Avoidance [19]. Furthermore, Pongsakornsathien et al. [20] proposed a data-driven framework to protect commercial UAM from cyber attacks and theft. The framework employs supervised learning-based models, such as decision trees, random forests, logistic regression, K-nearest neighbors (KNNs), and long short-term memory (LSTM), to predict and detect network interference and spoofing attacks. The proposed framework was validated using an updated drone attack dataset, demonstrating its efficacy in interference and spoofing detection with an accuracy of about 99.9% compared to decision trees, random forests, and KNNs, while also effectively identifying the root cause of the attack. These examples illustrate how the efficiency, interconnectivity, reliability, and flexibility of IT can contribute to various aspects of the UAM system, showcasing its unique potential in this field.

On the other hand, IT offers remarkable advantages for addressing other challenges related to future urban air traffic operations. Specifically, a large number of wireless communications are required in the UAM system for information exchange and control between aircraft, as well as for communication with ground equipment. These wireless communications necessitate the use of specific frequency bands and frequencies to ensure reliable and secure information transmission [21,22]. Han et al. [23] proposed the use of deep reinforcement learning (DRL) techniques for dynamic spectrum management in cellular-based urban air mobility (cUAM) . They defined an MDP for dynamic spectrum sharing in cUAM and adopted the deep Q-learning algorithm for training the agent. Their solution aims to minimize the total UAM mission completion time while ensuring reliable air-ground communication between the aircraft and its associated ground-based air traffic control center.

In conclusion, despite the vast variety and extensive applications of IT in the field of UAM, it remains technically immature and has not made full advantage of IT. Meanwhile, there is a notable lack of comprehensive reviews analyzing the connection and development between IT and UAM. Our objective is to address this gap and provide readers with a thorough understanding of the progress and accomplishments of IT in the UAM sector. This overview aims to synthesize the existing literature on IT's contributions to UAM development in order to elucidate its impact on this burgeoning field. Our primary contributions are as follows:

1. We summarize the development of IT technology in UAM and present a comprehensive collection of existing technological applications.
2. We provide a systematic overview of IT's role in various aspects of UAM, including vehicle design, flight path planning, safety, and security.
3. We identify the major challenges in applying IT to UAM and suggest corresponding future research directions to advance the field.

The remainder of this review is structured as follows: Section 2 offers an overview of the UAM system, primarily focusing on its components and the interactions between them. Section 3 specifically discusses the application of IT within the UAM system, along with a detailed classification of its development. Section 4 explores the future trends of IT in the UAM system and the outlook for UAM as a whole. In the final section, we present our conclusions and provide additional commentary.

## 2 UAM system overview

**Fig. 2:**
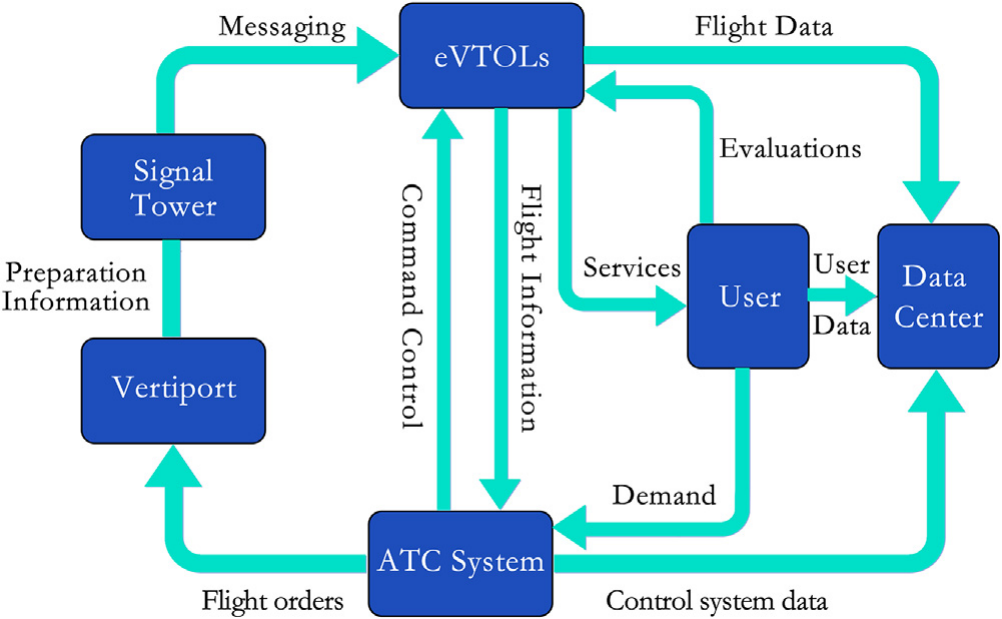
**UAM system composition and interaction between components**.

Urban traffic congestion has long been a pressing issue worldwide, prompting an urgent call for and growing interest in the UAM system [24]. Comprising multiple components and technologies that interact in complex ways, the UAM system is essential for achieving efficient and safe urban air traffic, positioning them as a crucial part of the future of urban air travel (see Fig. 2). In this paper, we will discuss the various components of a UAM system and how they collaborate to enable efficient and safe urban air mobility.

### 2.1 Electric vertical takeoff and landing aircraft

One of the most important components of UAM is electric Vertical Takeoff and Landing (eVTOL) aircraft, which are capable of taking off and landing vertically. Palaia et al. [25] presented a new conceptual design methodology for urban aircraft, tailored explicitly for eVTOL. This method considers many major aspects of aircraft design, including aerodynamics, mission analysis, structural design, and propulsion sizing, while also analyzing the sensitivity of different main design parameters and top aircraft requirements. eVTOL aircraft have a lot more advantages over conventional aircraft, as also pointed out in a review by Inwala and Parikh [26] that summarizes the various types and merits of eVTOL aircraft. The foremost strength of eVTOL aircraft is that they have no requirement for takeoff and landing facilities, such as a runway demanding a considerable amount of space, but a small platform. As a result, the need for extensive land and infrastructure is eliminated, and better maneuverability can be obtained. Also, eVTOL aircraft enable faster point-to-point services, potentially reducing travel time and reducing road congestion. Furthermore, the electrification, low noise, and zero emissions of eVTOL aircraft make them an environmentally friendly, low-carbon transportation option. Li [27] developed a physics-based prediction model for evaluating rotorcraft broadband noise. Additionally, a machine learning-based prediction model for rotorcraft trailing edge broadband noise was also developed using artificial neural networks (ANN) and linear regression. The ANN model is capable of accurately capturing the variation in noise levels, while the linear regression model is able to predict the general trend in noise levels.

### 2.2 Vertiports

Vertiports are crucial infrastructure for the UAM system, serving as the landing and docking points for eVTOL aircraft [28]. They function in a similar way as airports and train stations, providing the necessary space and facilities for the UAM system. In urban areas with limited space and high traffic congestion, it is important to establish sufficient Vertiports and carefully plan their sites in order to allow efficient and sustainable UAM system operations. Fadhil et al. [29] highlighted the importance of building ground infrastructure for the UAM system and suggested that Geographic Information System (GIS) based software can help effectively validate the placement sequence of Vertiports. Jeong et al. [30] utilized established algorithms and real-world data to select Vertiport locations based on the commuting population. Specifically, the K-means clustering was adopted to identify population clusters and the barycenters are selected as Vertiport locations. Also, a “noise-first” route is then created to minimize the number of people affected by noise.

### 2.3 Air traffic control system

As part of the urban air system, UAM must be coordinated and integrated with the existing Air Traffic Control (ATC) system to ensure safe and efficient operations [31]. The ATC system has a significant impact on the scheduling of eVTOL aircraft within the UAM system [32]. The integration of UAM into the urban air system requires an orderly air traffic environment, which is facilitated by the ATC system. The ATC system plays a crucial role in monitoring and dispatching urban air traffic to prevent collisions and conflicts between aircraft, ensuring the safety of UAM flights [33]. Moreover, by guiding the flight path and altitude of UAMs, the ATC system can optimize the flow of urban air traffic and improve traffic efficiency. Given the dynamic and complex nature of UAM operations, integrating UAM into the ATC system can help ensure safe and efficient transportation [34]. Therefore, given the constantly evolving nature of UAM, continuous technological innovation and optimization of the ATC system are crucial to ensure safe and efficient UAM operations. Deep reinforcement learning was employed by Kumar et al. [35] for the development of an ATC system that allows for eVTOL aircraft management at Vertiports. In their system, proximal policy optimization (PPO) algorithm is applied to learn Vertiport air traffic control policies. Additionally, a graph convolutional network is utilized to abstract the Vertiport space and the eVTOL space into graphs and aggregate information for a centralized ATC agent to generalize the environment.

### 2.4 User terminals

User terminals serve as a vital link between eVTOL service providers and end users, making them also an essential part of the UAM system. These devices, including mobile apps, smartphones, and tablets, enable users to access the UAM system and receive the necessary eVTOL services. User terminals play a crucial role in improving UAM acceptance and usability by facilitating better communication and interaction between service providers and end users, as well as supply chain and delivery management. To meet the changing needs and capabilities of the eVTOL system, user terminals must be customizable, user-friendly, and secure. A research platform presented by Dao et al. [36] provides a starting point for UAM operations research and development. The platform features a graphical user interface to support small eVTOL system operations for the Unmanned Aircraft Traffic Management (UTM) program. The UTM architecture lays a foundation for UAM research and development due to the operational similarities between UAM and UTM. The platform can also be used to test and evaluate different UAM concepts, including air cab services, and to develop new technologies and operating procedures to support safe and efficient UAM operations in metropolitan areas. As eVTOL technology and the UAM system continue to evolve, user terminals will become increasingly intelligent and diverse, enabling them to meet changing needs and challenges of the industry.

## 3 Application of IT in UAM

**Fig. 3:**
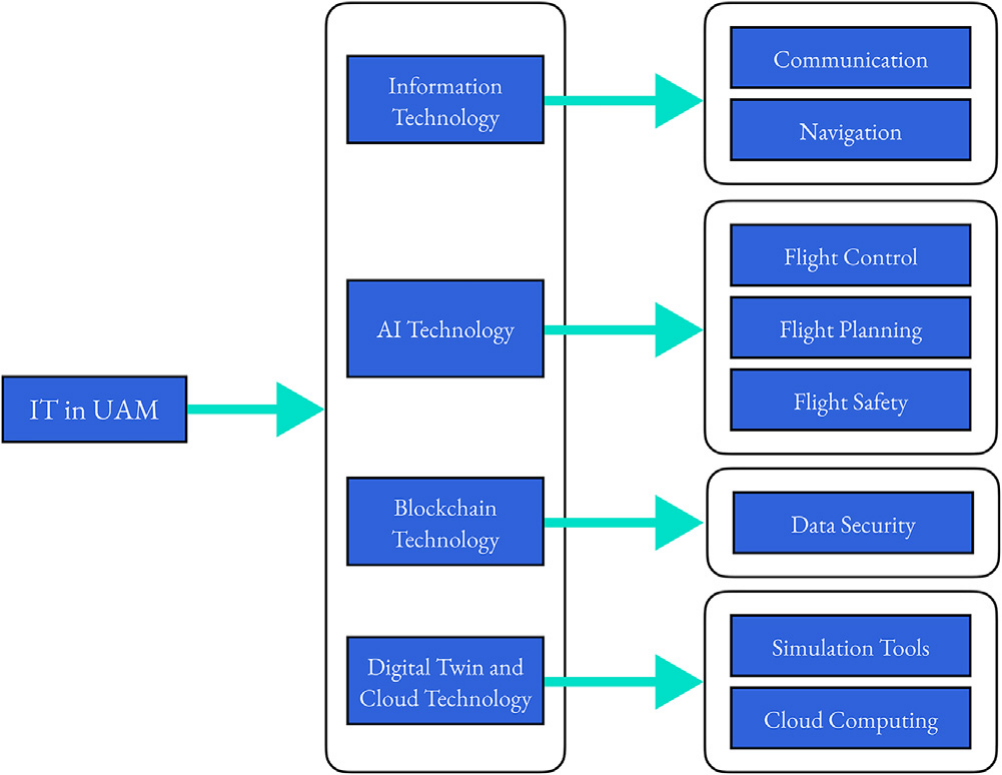
**Key IT technology to UAM development**.

In recent years, IT has undergone significant progress and development, driven by the widespread availability of high-speed internet, powerful computing systems, and advanced software applications [37,38]. IT has become an indispensable part of our daily lives. As a new urban transportation mode, UAM requires the support and assurance of IT for its design, operation, and safety. Fig. 3 outlines some of the key IT technologies that are crucial for the development of UAM. Notably, mainstream IT, such as AI and blockchain, have the potential to significantly enhance the intelligence and security of the UAM system [39]. These advancements in turn promote the application and development of UAM in cities. In this section, we will explore the specific applications of various IT technology in UAM, examining their impact on urban transportation. By leveraging these technologies, the UAM system can deliver more efficient, reliable, and safe services to meet the demands of modern urban mobility. A summary of IT technologies and their applications are provided in Table 1, which will be elaborated on in the following subsections.

**Table 1:** **Summary of IT technologies in UAM**.

IT Technology	Application	Category	Research
Information Technology	Communication Navigation	4G/5G networks DSRC 6G networks Vision-based navigation Navigation augmentation	Gheorghisor et al. [42], Park et al. [45,43] Dowd et al. [44] Shrestha et al. [46], Kim and Kim [47] Ye et al. [48] Bijjahalli et al. [49]
AI Technology	Flight control Flight planning Flight safety	Machine vision Speed recommendation Path planning Flow optimization Collision avoidance	Huang [50] Deniz and Wang [51] Dai et al. [52], Tang et al. [53] Sinha and Dipro [54] Bertram et al. [55]
Blockchain Technology	Data security	Permissioned blockchain Smart contract	Freeman and Garcia [56] de Oliveira et al. [57] Alkadi and Shoufan [58]
Digital Twin and Cloud Technology	Simulation tools Cloud computing	Digital twin Agent-based simulation Cloud-based management system	Brunelli et al. [59] Rothfeld et al. [60] Wieland et al. [61]

### 3.1 Information technology

Information technology plays a significant role in the development and operation of the UAM system. It enables the exchange of information between various system components, including eVTOL, ground control stations, and passengers. Communication and navigation are two key applications of information technology in UAM [40,41]. Effective communication is essential to the safe and efficient operation of UAM, and the UAM system uses a variety of communication technologies, such as 4 G/5 G networks, satellite communication, and dedicated short-range communication [42], [43], [44]. Park et al. [45] proposed several enhancement schemes for cellular networks to support UAM communications, using coupling loss and geometric SINR as two performance metrics to evaluate the link performance of UAM. The proposed solution involves the generation and deployment of over-the-air cells to support UAM communications, which can be managed and flexible depending on the location or density of the base stations. The solution also allows for the possible reuse or sharing of base stations between terrestrial and UAM communications. On the basis of the 5G communication technology, the application of 6G technology was also envisioned for UAM communication [46]. Kim and Kim [47] further discussed the future prospects of a 6G-enabled UTM ecosystem in a very dense urban air traffic scenario, focusing on non-ground functions, including air and satellite communications. Several urban airspace divisions are also presented, and a strategic management framework for dynamic airspace traffic management and conflict-free operations is discussed. The paper also provides a comprehensive comparison of existing schemes related to cellular technology-based airborne vehicle communication and their advantages and disadvantages. The author also provide an advanced study of an airborne experimental and research platform for an advanced radio infrastructure based on 5G and Beyond 5G communications, providing a roadmap for the development of a fully autonomous 6G-based UAM system that can operate safely and effectively in complex urban airspace environments.

Navigation technology is critical to UAM, and the UAM system uses a combination of satellite navigation systems like GPS, as well as inertial navigation systems and visual navigation systems. In light of the fact that the performance of satellite navigation system will degrade in dense urban environment, Bijjahalli et al. [49] investigated the impact of urban structures via explicit error analysis. Based on the predicted navigation performance, a guidance strategy is designed accounting for factors including elevation and signal reflection, in order to improve the safety and reliability of UAM. Ye et al. [48] presented two approaches for navigation in the final approach phase of eVTOL aircraft in UAM scenarios. The first method is a common hybrid navigation system, which integrates the satellite and inertial navigation system to provide accurate position information and vertical altitude guidance during the final approach phase. The integration of these two systems helps to improve the accuracy and reliability of the navigation system. The second approach is a vision-based stand-alone navigation system that uses camera images to gain extra position information. The camera parameters can be calibrated using a checkerboard pattern. The vision-based navigation system works independently and is not integrated with the system.

### 3.2 AI technology

AI technology has significantly transformed traditional ground transportation, with innovations like autonomous driving and in-vehicle networks being notable examples [62], [63], [64]. Its integration into the UAM systems has emerged as a pivotal factor, particularly in the control of eVTOL flight systems. This allows for enhanced autonomy, boosting flight safety and operational efficiency [65], [66], [67], [68].

By modeling eVTOL and optimizing control strategies, AI enables eVTOL aircraft to autonomously adjust flight controls based on real-time situations [69]. Huang [50] managed to improve the intelligent control of eVTOL by using machine vision combined with multi-agent decision-making techniques. The machine vision technique is improved by adding scale information to the least significant digit algorithm, which streamlines operational efficiency and trims down the computational demands of the eVTOL control system. Furthermore, it employs a multi-line segment criterion to merge candidate line segments, thus optimizing identification performance. Deniz and Wand [51] proposed a novel multi-agent reinforcement learning method that provides speed recommendations to eVTOL aircraft approaching urban air intersections, thus avoiding possible collisions and improving operation efficiency. This also helps reduce delays caused by traffic congestion and saves travel time through guidance provision for eVTOL aircraft to handle high-density UAM operations. Simulation results also demonstrated the effectiveness of this method in solving the intersection separation problem and improving ATC in urban airways.

AI's role is also pivotal in the flight planning of eVTOL, as it can optimize path planning, reduce travel time and enhance the passenger experience. Dai et al. [52] developed a conflict-free A* algorithm for four-dimensional path planning of UAM operations. The algorithm generates the shortest time-of-flight path while resolving conflicts with both static and dynamic obstacles, and numerical simulations showed that it can generate paths that resolve a large number of potential conflicts in airspace utilization with acceptable computation time and flight delay. Additionally, an automated flight planning system (AFPS) for UAM operations was proposed by Tang et al. [53]. This system features a Low-Altitude Airspace Management System (LAMS) that uses LiDAR data to produce a 3D map. From this map, a network is built using the visibility graph method to determine 3D shortest paths. Concurrently, the Low-Altitude Traffic Management System (LTMS) develops pre-departure 4D trajectories that are conflict-free, considering both operational costs and equity among operators. A specific flight-level assignment strategy is incorporated to address trajectory deconfliction in the LTMS, and a Nash social welfare program is integrated to ensure fairness among UAM operators.

Finally, AI technology has the potential to support the flight safety of eVTOL. Safety remains paramount for eVTOL flight, since UAMs frequently traverse urban zones, sometimes sharing spaces with ground vehicles and pedestrians. Sinha and Dipro [54] proposed a multi-criteria traffic clustering approach for optimizing traffic flows to support decision-marking in eVTOL traffic management and UAM operations. An on-site validation integrating the eVTOL system and UAM traffic data demonstrated its feasibility and efficiency in supporting the technical verification of future air traffic concepts like urban air cabs or delivery drones. Bertram et al. [55] utilized Monte Carlo Tree search to address aircraft collision avoidance. This approach is designed to navigate with collision avoidance when confronted by a large number of aircraft and obstacles, while maintaining sufficient efficiency to operate on low-power, lightweight embedded computing hardware used in avionics with limited processing power, memory, and storage capacity. Various strategies were explored to ensure the tractability of the problem tractable as well as scalability to larger number of aircraft.

### 3.3 Blockchain technology

Blockchain technology can provide a secure and decentralized platform for storing and exchanging UAM data without the need for a central authority, ensuring that sensitive information is protected from unauthorised access [70,71]. It can enhance data security and improve traffic management in the UAM system by providing real-time tracking of UAMs and their flight paths, allowing for efficient and secure management of UAM traffic [52,72]. Freeman and Garcia [56] proposed to address cybersecurity threats in UAM environments using a permissioned blockchain approach, which restricts network participation to authorized entities, thus ensuring data security and tamper-proofness. It makes the blockchain technology an effective solution for addressing cybersecurity threats, including man-in-the-middle attacks, spear phishing, and exploitation of valid accounts and public-facing applications. In addition, it is argued that the UAM architecture should be based on unmanned traffic management operations, where UAM operators, Providers of Services for UAM (PSUs), and Supplemental Data Service Providers (SDSPs) can use the blockchain network to securely exchange data and communicate with each other. PSUs can provide services such as airspace management, vehicle tracking and communication services, while SDSPs can provide additional data services such as weather information and traffic data. This will enable UAM operators to work together to manage aircraft in urban environments securely.

Beyond data security in the UAM system, blockchain technology can also support features like smart contracts. These contracts facilitate automated execution, performing traffic management tasks when certain conditions are met. Alkadi and Shoufan [58] explored the challenges of managing increasing air traffic due to the expansion of advanced air mobility (AAM). To address the challenge, they introduced a technique that leverages a secure, distributed approach to handle the intricate nature of AAM traffic. Specifically, this method employs trusted data structures and smart contracts within a blockchain environment to facilitate distributed airspace allocation management and conflict resolution. It not only minimizes the risk of system failures from single points of vulnerability but also incorporates a priority system to maintain fairness in airspace use.

### 3.4 Digital twin and cloud technology

Digital twin is an emerging technology that offers unique advantages in areas such as the industrial Internet, the energy industry, logistics and supply chain management, and is also increasingly being used in the UAM system [73], [74], [75]. With the digital twin technology, virtual copies of the UAM system can be created, allowing developers to simulate system behavior and performance before being built. This enables potential problems to be identified and resolved before they arise, ensuring the efficient and effective development of the UAM system. To integrate UAM services into existing transportation systems, Brunelli et al. [59] proposed an approach that involves the use of a digital twin model of the urban environment to simulate and analyze UAM scenarios. They also demonstrated a 3D spatial network model using a real-world scenario in the city of Bologna, Italy, showing the feasibility of using a digital twin model and 3D air network to determine safe and efficient flight paths for autonomous vehicles in urban environments. This approach provides a good way to explore the integration of UAM services into realistic environments. An agent-based extension for eVTOL service was developed by Rothfeld et al. [60] on the basis on MATSIm, which is capable of offering detailed comparison of congested travel times for traditional transport modes, such as car and public transport, versus the emergent UAM. Case study based on this tool support the potential travel time reduction due to UAM, and it accentuate the efficacy of agent-based modeling in understanding the dynamics of novel transport systems.

Cloud computing technology has a significant impact on the development and implementation of the UAM system, which require the processing of large amounts of data, including real-time aircraft positions, traffic control, weather conditions and sensor data. Cloud computing technology can provide efficient data storage and processing capabilities to support the UAM system. Wieland et al. [61] explored the concept of a cloud-based flight management system (CFMS) and its potential applications in UAM operations. The CFMS is the digital twin of the FMS, hosted in a cloud computing environment, which can access vast amounts of information that are not typically available to ground-based aviation systems, allowing the computational power that were previously impractical. The CFMS has many potential applications, including trajectory negotiation, exchange of rerouting information, exchange of city wind information, and handling of unplanned access to controlled airspace. Additionally, the CFMS has the potential to facilitate the quantification of concepts through laboratory simulation experiments and field flight tests.

## 4 Challenges and outlook

While the application of IT has brought about significant advances in the UAM system, there are still challenges that must be addressed to fully realize the potential of these technologies. This study highlights the challenges and difficulties encountered in implementing IT for UAM and identifies the following six research topics prospective for future investigation:

### 4.1 eVTOL aircraft

As an important part of the UAM system and one of the key modes of future on-demand transportation systems, eVTOLs are receiving increasing attention. At the same time, many technical and hardware limitations still need careful consideration in practical applications [76]. For example, the safety and reliability of UAVs, especially those relying on IT systems for autonomous decision-making, must be carefully evaluated to ensure passenger safety [77]. Moreover, the battery life and charging infrastructure of eVTOLs need to be considered to meet daily transportation needs [78]. Yang et al. [79] discussed the challenges and key requirements of batteries for eVTOLs. The unique operating conditions and requirements of eVTOLs present substantial difficulties for batteries, including the need for high specific energy and power, rapid recharging, long cycle life, and stringent safety standards. Addressing these challenges requires continuous technological advancements and infrastructure upgrades, and these issues will remain an ongoing topic of discussion in the future.

### 4.2 Noise pollution

In addition to the electrification of eVTOL aircraft, noise pollution during flight is another major challenge faced by the design of UAVs [80]. While UAV noise levels are generally lower than those of other transportation modes, they can still have a negative impact on the living quality of residents in densely populated urban areas. In particular, the noise generated during takeoff, landing, and flying-over can disturb the sleep and rest of surrounding residents, leading to physical and mental health problems, as well as reduced work efficiency. Therefore, noise control of eVTOL should be an important aspect of eVTOL under consideration. Afari and Mankbadi [81] developed an active noise control technology to reduce the in-plane thickness noise associated with multi-rotor advanced air mobility vehicles. Noise reduction technologies, such as low-noise propellers and sound-absorbing materials, should be developed and implemented to mitigate the impact of eVTOL noise on urban communities [82,83].

### 4.3 Integration and interoperability

Integration and interoperability, referring to the ability of effective communication and cooperation between different systems or components, are essential for the efficient operation of the UAM system. In smart urban transportation, various technologies and systems, including ground-based traffic management systems, urban air traffic systems, and individual mobility, must be synthesized to guarantee traveling efficiency [84], [85], [86]. Tuchen [87] discussed the evolving role of aviation in seamless, end-to-end multimodal transportation with the emergence of new airline market entrants such as UAM. Previous research efforts mainly focused on ground-based urban transportation, ignoring the increasing role that aviation will play in the future [88]. Thus, the author proposed a conceptual data model for integrating UAM with multimodal transportation, providing the basis for future UAM integration. In addition, the integration of diverse components of the UAM system can be challenging, requiring a high degree of interoperability between different technologies and components [89]. Interoperability is crucial as different components may be developed by different companies or organizations using different technologies and standards. Thus, it may be difficult for these components to communicate and cooperate effectively, leading to inefficiencies and delays in the transportation system. To address these interoperability issues, it is necessary to establish common standards and protocols for communication and data exchange between different components of an intelligent air traffic framework [90]. Developing standardized interfaces and data formats that allow different systems to communicate seamlessly with each other can facilitate interoperability [91].

### 4.4 Wireless connectivity performance

High-frequency data exchange and communication is another crucial requirement for the safety and operation efficiency of the UAM system.

On top of them, the performance of wireless connectivity for ground communication also influences the reliability, management, as well as maintenance of the system [92]. Zeng et al. [93] developed a spatial model based on stochastic geometry to describe the performance of wireless connectivity in UAM. The simulation results provide practical UAM design guidelines by showing the connectivity performance under different parameter settings. Al-Rubaye and Tsourdos [94] posited a prospective use case for sixth-generation (6 G) communication technology buttressed by AI systems that could aid the safe and secure integration of UAM operations. This novel architectural design can facilitate sundry routine tasks across all airspace. Therefore, future research needs to consider the wireless connectivity performance for ground-to-ground communication to ensure that the communication requirements for the UAM system are met.

### 4.5 Communication security

Aside from communication quality, ensuring the confidentiality and security of sensitive information is critical in interconnected systems like the UAM system. Quantum communication is one technological advancement that has shown great potential in ensuring secure and reliable data transmission [95]. In the operation process of UAVs, their communication with ground infrastructure through open channels can be vulnerable to various security attacks. Therefore, a secure mutual authentication scheme is necessary for the UAM environment. Kwon et al. [96] designed a secure and efficient handover authentication scheme for the UAM system. This study demonstrates the importance of a secure mutual authentication scheme and the need for switching authentication to ensure seamless communication when the service location changes.

### 4.6 Spectrum resource management

Spectrum resources are another type of crucial component of the UAM system. Similar to wireless connection, reliable radio communications, which ensure secure and reliable transmissions through the radio spectrum, are required by UAM aircraft to support information exchange and control [97]. owever, the availability of spectrum resources in urban areas is relatively limited and crowded due to shared frequency bands with a wide range of devices [98]. This poses a significant challenge to UAM developers and operators, who need to find innovative ways to optimize spectrum usage and ensure reliable and secure communications between vehicles and other system components. The shortage of spectrum resources can hinder the widespread adoption of this promising transportation technology, without which the UAM system may face issues of safety and efficiency. Therefore, it is essential for stakeholders to work together to address this challenge and find solutions to ensure reliable and secure radio communications for the UAM system in urban environments [99].

## 5 Conclusion

In conclusion, the development of UAM relies heavily on IT, allowing for a wide range of applications, including air traffic management, flight control, flight safety, and data security. However, the UAM system is confronted with a number of challenges, including integration and interoperability, the endurance of the eVTOLs, noise pollution, as well as communication safety and efficiency. Addressing these issues is critical to realizing the full potential of eVTOLs and ensuring their successful integration into existing urban transportation systems. In spite of these issues and challenges, eVTOLs enjoy a promising future and are expected to play an essential role in meeting the growing demand for urban transportation in the coming decades. Continued research and development of the UAM system, along with collaboration among stakeholders, is critical to the success of this transformative transportation system.

## Declaration of competing interest

The authors declare that they have no conflicts of interest in this work.
